# Non-infectious inflammation in atrial septum after GORE® CARDIOFORM septal occluder implantation

**DOI:** 10.1093/ehjcr/ytaf171

**Published:** 2025-04-08

**Authors:** Ryotaro Maeda, Takeshi Nakamura, Michiyo Yamano, Satoaki Matoba

**Affiliations:** Department of Cardiovascular Medicine, Graduate School of Medical Science, Kyoto Prefectural University of Medicine, 465 Kajii-cho Kawaramachi-Hirokoji, Kamigyo-ward, Kyoto 602-8566, Japan; Department of Cardiovascular Medicine, Graduate School of Medical Science, Kyoto Prefectural University of Medicine, 465 Kajii-cho Kawaramachi-Hirokoji, Kamigyo-ward, Kyoto 602-8566, Japan; Department of Cardiovascular Medicine, Graduate School of Medical Science, Kyoto Prefectural University of Medicine, 465 Kajii-cho Kawaramachi-Hirokoji, Kamigyo-ward, Kyoto 602-8566, Japan; Department of Cardiovascular Medicine, Graduate School of Medical Science, Kyoto Prefectural University of Medicine, 465 Kajii-cho Kawaramachi-Hirokoji, Kamigyo-ward, Kyoto 602-8566, Japan

## Case description

**Figure 1:**
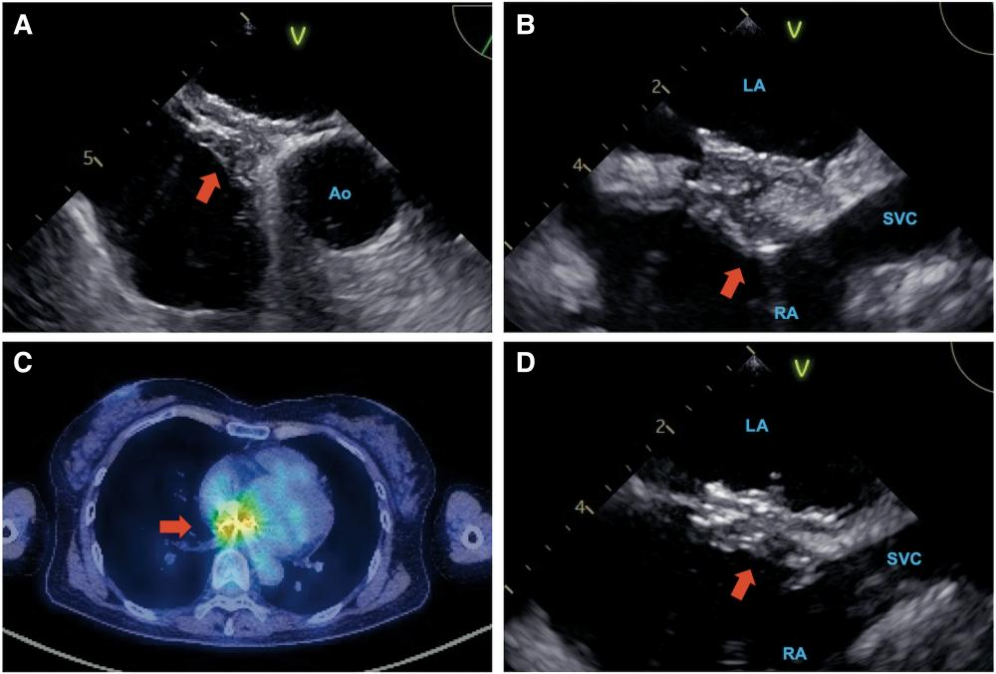
(*A* and *B*): (*A*) Mid-oesophageal aortic valve short-axis view, (*B*) mid-oesophageal bicaval view. The first transoesophageal echocardiography shows marked thickening of the atrial septal wall at the site of GORE CARDIOFORM septal occluder (GSO) implantation (arrow). (*C*) An ^18^F-fluorodeoxyglucose (FDG) positron emission tomography shows a high level of ^18^F-fluorodeoxyglucose accumulation at the site of the GORE CARDIOFORM septal occluder implanted in the atrial septum (arrow). (*D*) Mid-oesophageal bicaval view. The second transoesophageal echocardiography shows a remission of the atrial septal wall thickening at the site of the GORE CARDIOFORM septal occluder implantation (arrow). Ao, aorta; LA, left atrium; RA, right atrium; SVC, superior vena cava.

A 56-year-old female patient was admitted to our institution with fever 5 days after uncomplicated implantation of a 25-mm GSO device for patent foramen ovale which caused paradoxical cerebral infarction. Although the laboratory test showed a high level of inflammatory reaction (C-reactive protein: 9.45 mg/dL), repeated blood cultures and procalcitonin were all negative. Transoesophageal echocardiography demonstrated marked wall thickening of the atrial septum at and around the site of the GSO implantation (*Figure 1A* and *B*), though no other abnormal findings such as vegetation, intracardiac thrombus, pericardial effusion, or erosion were detected. An ^18^F-fluorodeoxyglucose positron emission tomography (FDG-PET) revealed a high level of FDG accumulation at the site of the GSO implanted in the atrial septum (*Figure 1C*). A patch testing for components of GSO [nickel, titanium, and expanded polytetrafluoroethylene (ePTFE)] was all negative. Her fever gradually subsided within 7 days of admission, and the laboratory test showed that her inflammatory response was decreasing day by day. The second TEE, 1 week after the first, demonstrated a spontaneous remission of the atrial septal wall thickening (*Figure 1D*). She was discharged after careful observation and had no recurrence of symptoms at her 1-month follow-up. Based on the results of each examination, the origin of the fever was considered to be a non-infectious, non-allergic inflammation caused by excessive healing process of the atrial septum. Patent foramen ovale closure using a percutaneous occluder is a well-established procedure, and complications requiring surgical explantation of a device due to infection, erosion, and allergy are rarely reported.^1,2^ However, the development of fever and inflammation after the procedure is sometimes observed, which resolves spontaneously.^3^ The present case suggests that close observation should be sought first instead of hasty explantation after exclusion of device infection, erosion, thrombosis, and allergic reaction.

##  

**Consent:** The authors confirm that written consent for the submission and publication of this case report has been obtained from the patient in line with the Committee on Publication Ethics guidance.

**Funding:** None declared.

## Data Availability

The data underlying this article are available in the article.
